# The effects of Ginsenosides on PI3K/AKT signaling pathway

**DOI:** 10.1007/s11033-022-07270-y

**Published:** 2022-02-27

**Authors:** Soudeh Ghafouri-Fard, Neda Balaei, Hamed Shoorei, Syed Muhammad Farid Hasan, Bashdar Mahmud Hussen, Seyedeh Fahimeh Talebi, Mohammad Taheri, Seyed Abdulmajid Ayatollahi

**Affiliations:** 1 Department of Medical Genetics, School of Medicine, Shahid Beheshti University of Medical Sciences, Tehran, Iran; 2 Department of Pharmacology, Tabriz University of Medical Sciences, Tabriz, Iran; 3 Department of Anatomical Sciences, Faculty of Medicine, Birjand University of Medical Sciences, Birjand, Iran; 4 Department of Pharmaceutics, Faculty of Pharmacy and Pharmaceutical Sciences, University of Karachi, Karachi, Pakistan; 5 Department of Pharmacognosy, College of Pharmacy, Hawler Medical University, Kurdistan Region, Iraq; 6 Department of Pharmacology, College of Pharmacy, Birjand University of Medical Sciences, Birjand, Iran; 7 Institute of Human Genetics, Jena University Hospital, Jena, Germany; 8 Phytochemistry Research Center, Shahid Beheshti University of Medical Sciences, Tehran, Iran

**Keywords:** Ginsenoside, Gene expression, Signaling pathway

## Abstract

Ginsenosides belong to a group of steroid glycosides that are extracted from the plant genus *Panax* (ginseng). This plant has been used for a long time for the treatment of a variety of disorders in traditional medicine. Recent studies have assessed the biological impact of Ginsenosides in cell culture or animal models. Animal studies have shown their beneficial impacts in the remedy of pathological conditions in different tissues. The ameliorating effects of Ginsenosides in diverse pathogenic conditions can be attributed to their effects on the production of reactive oxygen species. These substances mainly affect the activity of AMPK/AKT and PI3K/AKT pathways. The beneficial effects of Ginsenosides have been appraised in diabetes-related complications, spinal cord injury, cerebral ischemia, myocardial ischemia, and other disorders which are associated with oxidative stress. Moreover, these substances have been shown to interfere with the pathologic conditions during carcinogenesis. In the current study, we explain these impacts in two distinct sections including non-neoplastic conditions and neoplastic conditions.

## Introduction

**Fig. 1:**
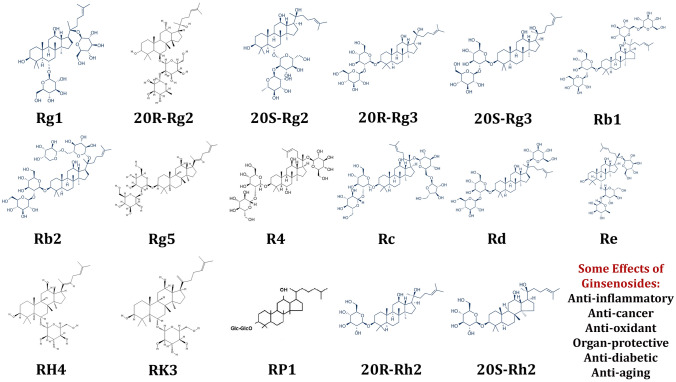
Chemical structure of some ginsenosides studied in this paper. Ginsenosides are the major constituents found in the plant ginseng. It has been reported that they have unique biological activities such as anti-aging, anti-oxidant, anti-tumor, anti-diabetic, and organ-protective impacts

Ginsenosides are a group of steroid glycosides and triterpene saponins being extracted from the plant genus *Panax* (ginseng). This plant has been used in traditional medicine for a long time. Ginsenosides have a great diversity of delicate and complex biological impacts when assessed separately [1]. Ginsenosides have been extracted from several parts of the plant, although usually from its roots. The purification process can be accomplished using column chromatography [2]. Asian, American, and Japanese species have distinctive chemical features. Asian ginseng (*Panax ginseng*) is the most extensively studied species because of its use in the traditional Chinese medicine. The majority of known ginsenosides belong to the dammarane family, based on the presence of a 4-ring, steroid-like configuration. A minimum of 2 or 3 hydroxyl groups are attached at the carbon-3 and -20 positions of the ginsenosides or their carbon-3, -6, and -20 positions, respectively. In protopanaxadiols and protopanaxatriols, sugar groups are bound to the carbon-3 and carbon-6 positions, respectively. Rb1, Rb2, Rg3, Rh2, and Rh3 are the most studied protopanaxadiols, while Rg1, Rg2, and Rh1 are famous protopanaxatriols (Fig. 1) [3].

The biological impact of ginsenosides has been assessed in cell culture or animal models. Animal studies have shown their beneficial impacts in the remedy of pathological conditions in different tissues. In the current study, we explain these impacts in two distinct sections including non-neoplastic conditions and neoplastic conditions.

## Non-neoplastic conditions

### Diabetic complications

**Fig. 2:**
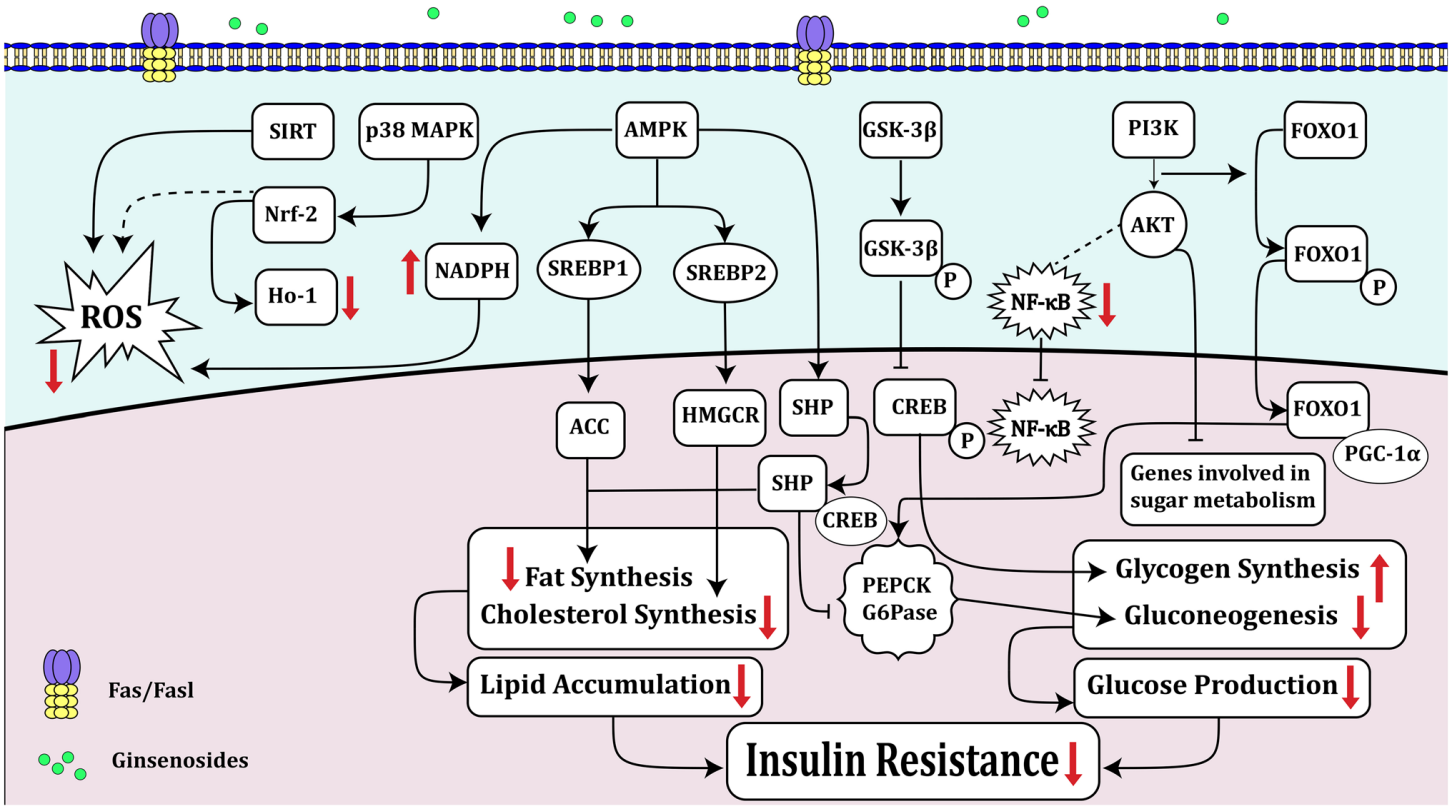
Ginsenosides can affect the activity of PI3K/AKT, GSK-3β, and AMPK pathways resulting in decreasing oxidative stress [4], inflammation, gluconeogenesis, glucose production, lipid accumulation, as well as insulin resistance [12]. There are three types of AKT substrates, GSK-3β, FOXO1, and PGC-1α, that could be involved in liver glucose production. The activated PI3K/AKT pathway could participate in insulin metabolism and glucose uptake improvement via translocating GLUTs (glucose transporters, especially GLUT4) to the cell membrane and/or through enhancing glycogen synthesis that happens by phosphorylation of glycogen synthase kinase 3 (GSK-3β) [12, 13]. On the one hand, FOXO could regulate insulin responsiveness and glucose homeostasis [12]. Moreover, AMPK could decrease fat and cholesterol synthesis. Ginsenosides can also decrease insulin resistance. In addition, ginsenosides can inhibit ROS production via activating Nrf-2, HO-1, and AKT [14–16]. They can also inhibit the NF-кB signaling pathway and decrease inflammation via blocking the mRNA expression of pro-inflammatory mediators as well as cytokines, including TNF-α, IL-1β, iNOS, and COX-2 [14]

The impact of Ginsenoside Rb1 in the amelioration of oxidative stress has been assessed in a bone marrow cell line obtained from a patient with neuroblastoma (SH-SY5Y) following treatment with a highly reactive metabolite of hyperglycemia, namely methylglyoxal (MGO). Ginsenoside Rb1 has been shown to alleviate the effects of MGO on the activity of superoxide dismutase and catalase and the level of total glutathione. This substance has decreased malondialdehyde levels, amended mitochondrial injury, and reduced production of reactive oxygen species (ROS) (Fig. 2). Besides, this substance has enhanced the Bcl-2/Bax ratio, decreased levels of cleaved caspase-3 and cleaved caspase-9, and increased phosphorylated AKT levels. Notably, the ameliorative impact of Ginsenoside Rb1 against MGO-associated apoptosis has been partially obliterated by an inhibitor of PI3K phosphorylation, implying that Ginsenoside Rb1 amends MGO-associated oxidative stress and apoptosis through enhancing the activity of PI3K/AKT cascade [4].

Ginsenoside Re has been shown to amend high glucose (HG)-induced injury in the retinal endothelial cells through modulation of the effects of PI3K/AKT cascade on HIF-1α/VEGF signaling. Cell line studies have shown that pre-treatment of these cells with Ginsenoside Re has protected these cells from HG-induced injury, decreased their apoptosis, and reduced ROS production. Ginsenoside Re has also enhanced the expression of HIF-1α in the cytoplasm but reduced its nuclear levels, implying that this substance decreases nuclear translocation of HIF-1α and decreases VEGF level. These effects are exerted through enhancing the activity of the PI3K/AKT pathway since they have been abrogated by a specific PI3K inhibitor. Thus, Ginsenoside Re affects the activities of PI3K/AKT and HIF-1α/VEGF pathways. These effects might be associated with the amelioration of HG-associated retinal angiogenesis [5].

Ginsenoside Rg1 has been shown to interfere with the effects of tau hyperphosphorylation on diabetic synaptic neurodegeneration of retinal ganglion cells, an early event in the pathogenesis of diabetic retinopathy. The neuroprotective impact of Ginsenoside Rg1 on diabetic retinae has been abolished after the suppression of expression of IRS-1 or AKT. On the other hand, suppression of retinal GSK3β has rescued the neuroprotective effects of Ginsenoside Rg1 when AKT was inhibited. Thus, Ginsenoside Rg1 can stop hyperphosphorylated tau-associated synaptic neurodegeneration of retinal ganglion cells through enhancing the activity of IRS-1/AKT/GSK3β cascade [6]. Table 1 lists the beneficial effects of Ginsenosides in diabetic complications.

**Table 1:** Ginsenosides effects on diabetic complication

Type of Diseases	Samples	Cell Lines	Ginsenoside	Dose range	Target	Pathway	Function	Ref
Diabetic Encephalopathy (DE)	In vitro	SH-SY5Y	Rb1	0–12 µM	Bcl-2, Bax, Caspase-3/9	PI3K/AKT	Ginsenoside Rb1 via activating the PI3K/AKT pathway could mitigate apoptosis and oxidative stress induced by MGO (Methylglyoxal) in SH-SY5Y cells.	[4]
Diabetic Retinopathy (DR)	In vitro	RF/6A	Re	0–10 µM 0–10 µM	HIF-1α ,VEGF, Caspase-3/9	PI3K/AKT	Ginsenoside Re via regulating the PI3K/AKT inhibits HIF-1α/VEGF signaling and attenuates high glucose (HG)-induced RF/6A injury.	[5]
DR	In vivo (Mouse)	Retinal ganglion cells	Rg1	2.5–10 µM	IRS-1, GSK-3β	AKT	Ginsenoside Rg1 via activating the IRS-1/AKT/GSK-3β axis could suppress hyperphosphorylated tau-triggered diabetic retinal neurodegeneration in mice.	[6]
Diabetic Nephropathy (DN)	In vivo (Rat)	-	Rg1	0–50 µM	Nephrin, α-SMA, GSK-3β, β-catenin	AKT	Ginsenoside Rg1 via the AKT/GSK-3β/β-catenin pathway could improve the tissue function of DN in rats.	[7]
Diabetes	In vivo (Mouse), In vitro	3T3-L1	Rb2	40 mg/kg, 1–25 µM	IKKβ, IκBɑ, IL-6, SOCS-3	PI3K/AKT	Ginsenoside Rb2 via the PI3K/AKT pathway could decrease the accumulation of fat and regulate the resistance of insulin.	[8]
Diabetes Mellitus (T2DM)	In vivo (Mouse), In vitro	HepG2	Rg1	50 mg/kg, 10 µM	PEPCK, G6Pase, FoxO1	AKT	Ginsenoside Rg1 via AKT/FoxO1 axis could decrease gluconeogenesis to the response of fasting hormone glucagon in T2DM.	[9]
T2DM	In vitro, In vivo (mice)	HepG2	Rk3	0.1–0.3 µM 10-60 mg/kg	GLUT2, G6pase, PEPCK, TNF-α, IL-6, NF-kB	AMPK/AKT	Ginsenoside Rk3 via the AMPK/AKT pathways could improve insulin resistance (by reversing hepatic gluconeogenesis and reducing lipid accumulation)	[10]
Hepatic Insulin Resistance	In vitro	HepG2	Rg1	0–80 µM	PEPCK, G6Pase, IRS	PI3K/AKT	Ginsenoside Rg1 via the IRS/PI3K/AKT axis could increase the consumption of glucose and reduce the resistance of insulin in HepG2 cells.	[11]

### Central nervous system

In vitro studies have shown that Ginsenoside Rg1 induces remedy of the scratch wound via enhancing the production of laminin and fibronectin as well as several growth factors including NGF, GDNF, and bFGF. In addition, Ginsenoside Rg1 could activate the PI3K/AKT signaling and promote the functional remedy of hindlimb movements in animal models. This substance could also reduce the void area and decrease levels of glial fibrillary acidic protein (GFAP) and chondroitin sulfate proteoglycans. In brief, Ginsenoside Rg1 can both enhance the scratch wound remedy in cell cultures via induction of expression of neurotrophic factors for astroglial cells and improve the functional remedy in animal models of spinal cord injury (SCI) [17].

Ginsenoside Rg1 has been found to promote the migratory potential of olfactory ensheathing cells in vitro, and their remedial impact in the treatment of SCI. This substance has enhanced the expression of MMP-2, MMP-9, and NCAM1 genes in olfactory ensheathing cells. Ginsenoside Rg1 has enhanced the migration of olfactory ensheathing cells through the PI3K/AKT pathway. Pre-treatment of olfactory ensheathing cells Ginsenoside Rg1 has improved their therapeutic efficacy in a rat model of SCI [18].

Ginsenoside Rd via the PI3K/AKT/GSK-3β axis could decrease phosphorylation of tau protein after cerebral ischemia [19]. This substance could also improve neurogenesis after cerebral ischemia through the PI3K/AKT pathway [20]. Table 2 shows the effects of Ginsenosides in disorders of the central nervous system (CNS).

**Table 2:** Effects of ginsenoside in CNS disorders

Type of Diseases	Samples	Cell Lines	Ginsenoside	Dose range	Target	Pathway	Function	Ref
Spinal Cord Injury (SCI)	In vivo	Astrocytes	Rg1	40 µg/mL	bFGF, GDNF, NGF	PI3K/AKT	Ginsenoside Rg1 via the PI3K/AKT pathway could inhibit GFAP expression after SCI.	[17]
SCI	In vivo	OECs	Rg1	40 µg/mL	GDNF, BDNF, NGF, CNTF, VEGF, MMP-2/9, NCAM1	PI3K/AKT	Ginsenoside Rg1 via the PI3K/AKT pathway could promote olfactory ensheathing cell migration.	[18]
Cerebral Ischemia	In vivo (Rat)	-	Rd	30 mg/kg	PHF-1, GSK-3β	PI3K/AKT	Ginsenoside Rd via the PI3K/AKT/GSK-3β axis could decrease phosphorylation of tau protein after cerebral ischemia.	[19]
Cerebral Ischemia	In vivo (Rat), In vitro	SH-SY5Y	Rb1	25–100 mg/kg, 10 µmol/L	PTEN	AKT/mTOR	Ginsenoside Rb1 via the AKT/mTOR/PTEN axis could protect neurons in an artificial abnormal microenvironment.	[21]
Cerebral Ischemia	In vivo (Rat), In vitro	PC12	Rd	1–5 mg/kg, 50 and 100 µmol/L	VEGF, BDNF	PI3K/AKT	Ginsenoside Rd via the PI3K/AKT pathway could improve neurogenesis after cerebral ischemia.	[20]
Cerebral I/R Injury	In vitro	SH-SY5Y	Rb1	1-100 µmol/L	LC3I/II, Beclin-1	PI3K/AKT	GRb1 could mitigate OGD (oxygen-glucose deprivation)-induced autophagic vacuoles in SH-SY5Y cells.	[22]
Alzheimer’s disease (AD)	In vitro	PC12	Rg2	5–20 µg/mL	Bcl-2, Bax, Caspase-3	PI3K/AKT	Ginsenoside Rg2 via the PI3K/AKT pathway could protect PC12 cells against β-amyloid25-35 apoptosis.	[23]
Neurotoxicity	In vitro	PC12	R4	25, 50, and 100 µM	Caspase-3/8/9, Bax, GSK-3β	PI3K/AKT	Ginsenoside R4 via the PI3K/AKT/GSK-3β axis could reduce neurotoxicity pain in PC12 cells.	[24]
Neurotoxicity	In vivo (Mouse)	-	Re, Rg1 or Rb1	20 mg/kg	IL-6, IFN-γ, TNF-α, IL-1β, Bax, Bcl-2, Caspase-3	PI3K/AKT	Ginsenoside Re via the IL-6-mediated PI3K/AKT pathway could improve treatment neurotoxicity induced by TMT (trimethyltin).	[25]

### Cardiovascular disorders

Experiments in rat heart myoblasts have shown that hypoxia induces a reduction in cell viability and increases apoptosis and autophagy in these cells. Notably, Ginsenoside Rg1 has ameliorated hypoxia-associated changes in these cells without any impacts on their viability under normal oxygen concentrations. Ginsenoside Rg1 has enhanced phosphorylated levels of important kinases in the PI3K/AKT/mTOR pathway and levels of HIF-1α. Additional in vivo assays have confirmed the effects of Ginsenoside Rg1 in amelioration of ischemia/reperfusion injury in rats via enhancing the activity of the PI3K/AKT/mTOR pathway. Therefore, Ginsenoside Rg1 protects cardiomyocytes from hypoxia-associated cell damage by enhancing the expression of HIF-1α via activating the PI3K/AKT/mTOR pathway [26]. Another study has shown the effects of Ginsenoside Rg3 in the protection of cardiomyocytes against apoptosis in myocardial ischemia via modulation of the AKT/eNOS and Bcl-2/Bax pathways [27]. Ginsenoside Rg2 has also been shown to improve cardiac function and amend myocardial fibrosis following myocardial infarction. This substance has suppressed collagen deposition in mice following myocardial infarction. Moreover, Ginsenoside Rg2 has decreased expressions of Collagen I (Col 1), Col 3, and alpha-smooth muscle actin through enhancing the activity of phosphorylated AKT in angiotensin II-induced cardiac fibroblasts. Since ginsenoside Rg2 can emend heart function and decrease cardiac fibrosis, it might be a putative medication for the prevention of ventricular remodeling following myocardial infarction [28]. Table 3 shows the effects of Ginsenosides in the treatment of cardiovascular diseases.

**Table 3:** Effects of ginsenoside on cardiovascular diseases

Type of Diseases	Samples	Cell Lines	Ginsenoside	Dose range	Target	Pathway	Function	Ref
Myocardial Ischemia (MI)	In vivo (Rat), In vitro	H9c2	Rg1	10 mg/kg, 0-200 µM	HIF-1α, Bax, Bcl-2, Caspase-3/9	PI3K/AKT/mTOR	Ginsenoside Rg1 via the PI3K/AKT/mTOR pathway could protect against heart injury induced by hypoxia.	[26]
MI	In vivo (Rat)	-	Rg3	0.1–100 µM	Caspase-3/9, Bcl2, Bax, eNOS	AKT	Ginsenoside Rg3 via the AKT/eNOS and Bcl-2/Bax pathways could protect cardiac cells against apoptosis in MI.	[27]
MI	In vitro	H9c2	Rb1	3.125–12.5 µg/mL	Caspase-3/8/9, Bcl-2, Bax, Bid	AKT, JNK, ERK1/2	Ginsenoside Rb1 via the AKT, JNK and ERK1/2 pathways could inhibit apoptosis in cardiomyocytes.	[29]
MI	In vitro	NRCFs	Rg2	1-100 µM	Col1/3, α-SMA	AKT	Ginsenoside Rg2 via the AKT pathway could improve cardiac function after MI in NRCFs cells.	[28]

## Other non-neoplastic conditions

20(R)-Ginsenoside Rg3 has been shown to ameliorate acetaminophen-induced liver damage in animal models through activating PI3K/AKT cascade. Pretreatment of mice with a certain dose of this substance has attenuated the effects of acetaminophen on levels of ALT, AST, TNF-α, and IL-1β. Moreover, Ginsenoside Rg3 could ameliorate the effects of acetaminophen on GSH and MDA levels as well as up-regulation of CYP2E1 and 4-HNE. Pretreatment of animals with this substance has also alleviated acetaminophen-induced apoptosis, necrosis, and inflammatory infiltration in the hepatic tissue [30]. Ginsenoside Rg1 could prevent starvation-induced muscle protein degradation via regulating the AKT/mTOR/FoxO axis in C2C12 myotubes [31]. In addition, 20 (S)-ginsenoside Rg3 via regulating the AKT/mTOR/FoxO3 axis could protect against myotube atrophy [7]. Ginsenoside Rh2 could decrease inflammatory responses in the lung tissue and lung injury via PI3K/AKT/mTOR and MEK/ERK pathways [28]. On the other hand, Ginsenoside Rg1 via reducing the activity of the AKT/mTOR pathway could attenuate cognitive impairment and senescence of neural stem cells induced by D-galactose [32]. Table 4 shows the effects of Ginsenosides in the treatment of diverse pathological conditions.

**Table 4:** Effects of ginsenoside on other non-neoplastic conditions

Type of Diseases	Samples	Cell Lines	Ginsenoside	Dose range	Target	Pathway	Function	Ref
Acetaminophen Hepatotoxicity (APAP)	In vivo (Mouse)	-	20(R)-Rg3	10 and 20 mg/kg	Bcl-2, Bax, NF-κB	PI3K/AKT	20(R)-Rg3 via the PI3K/AKT pathway could improve APAP toxicity in the liver.	[30]
Skeletal Muscle Atrophy	In vitro	C2C12	Rg1	1-100 µM	Atrogin-1, MuRF-1, FoxO	AKT/mTOR	Ginsenoside Rg1 via regulating the AKT/mTOR/FoxO axis in C2C12 myotubes could prevent starvation-induced muscle protein degradation.	[31]
Atrophy	In vitro	C2C12 myoblasts	Rb1, Rc, Rb2, Re, Rb3, Rg1, S-Rg3, R-Rg3, Rd	0.02-2 µM	Myogenin, MyoD, MuRF1, Atrogin-1, FoxO3	AKT/mTOR	20 (S)-ginsenoside Rg3 via regulating the AKT/mTOR/FoxO3 axis could protect against myotube atrophy.	[7]
Muscular hypertrophy	In vitro	C2C12 myoblasts	Rb1, Rb2	0-100 µM, 0-100 µM	MyoD, E-protein, MHC, Myogenin	AKT/mTOR	Ginsenoside Rb1 and Rb2 via upregulating the AKT/mTOR pathway could increase myotube hypertrophy myoblast differentiation.	[33]
Lung Injury	In vivo (Mouse)	-	GRh2	5–20 mg/kg	NO, TNF-α, IL-1β , IL-4/6, TLR4, Raf-1, Keap1, Nrf2, HO-1	PI3K/AKT/mTOR, MEK/ERK	Ginsenoside Rh2 via the mentioned pathways could decrease inflammation of the lung in mice.	[34]
Acute lung injury (ALI)	In vivo (Mouse), In vitro	RAW264.7	Rg3	10–30 mg/kg, 25–100 µM	TNF-α, IL-1β, IL-6, IL-10, TGF-β	PI3K/AKT/mTOR	Ginsenoside Rg3 via the PI3K/AKT/mTOR pathway could reduce LPS inflammation in ALI.	[35]
Osteoarthritis	In vitro	Rat articular chondrocytes	Rg1	0.001-100 µg/ml	Bcl-2, Bax, Cytochrome-c, Caspase-3	PI3K/AKT	Ginsenoside Rg1 via the PI3K/AKT pathway could inhibit apoptosis in chondrocytes.	[36]
Intestinal I/R Injury	In vivo (Rat)	-	Rb1	0.6 and 15 mg/kg	MDA, SOD, TNF-α, IL-6, IL-1β	PI3K/AKT/Nrf2	Ginsenoside Rb1 via activating the PI3K/AKT/Nrf2 pathway could attenuate inflammation and oxidative stress in intestinal I/R injury.	[37]
Oxidative Stress (OS)	In vitro	293T	Rc	0–50 µM	MnSOD	AKT/FoxO1	Ginsenoside Rc by modulating the AKT/FoxO1 pathway could suppress OS.	[38]
Oxidative Stress (OS)	In vitro	hUCBDSCs	Rg1	0.01-50 µM	Caspase-3, Bim, Bcl-2, Bax, FoxO3a	AKT	Ginsenoside Rg1 via AKT/FoxO3a/Bim axis could increase survival of hUCBDSCs against tert-Butyl hydroperoxide (t-BHP) induce apoptosis.	[39]
Oxidative Stress	In vivo (Mouse), In vitro	NSCs	Rg1	20 mg/kg, 0–40 µg/mL	MDA, ROS, SOD, GSH-px	AKT/mTOR	Ginsenoside Rg1 via reducing AKT/mTOR pathway could attenuate cognitive impairment and senescence of neural stem cells induced by D-galactose.	[32]
Immunodeficiency Syndrome	In vitro	CHME5	Rb1	0–20 µM	PDK-1	AKT	Ginsenoside Rb1 by inhibiting the AKT pathway could eliminate HIV-1 (D3)-transduced cytoprotective human macrophages.	[40]
Bacterial infections	In vivo (Mouse), In vitro	RAW264.7	Rb1	0–5 mg/kg, 0–10 µM	-	p38 MAPK/AKT	Ginsenoside Rb1 through the p38 MAPK/AKT pathway could increase macrophage phagocytosis.	[41]

## Neoplastic conditions

### Leukemia

**Fig. 3:**
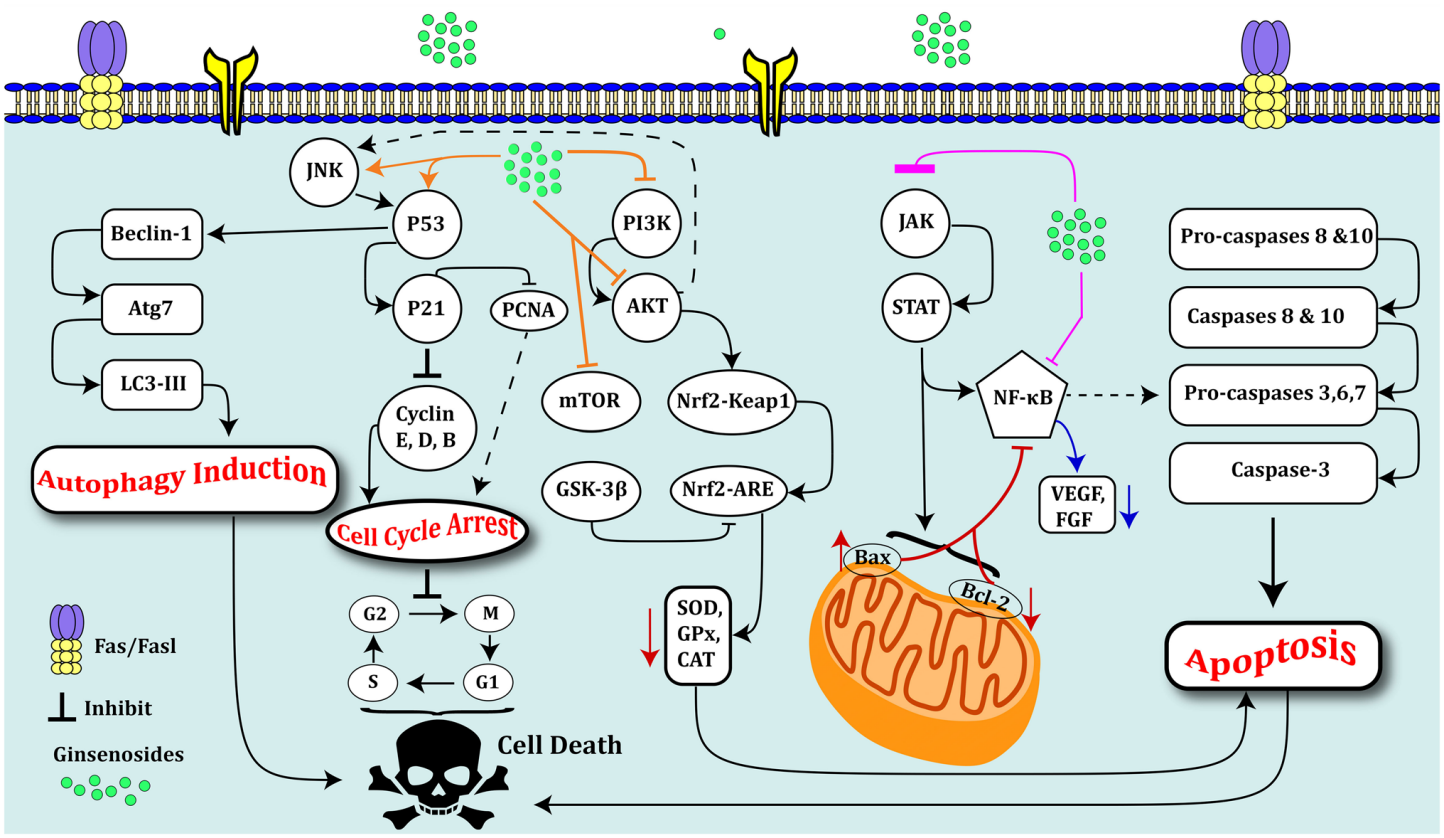
Several studies have shown that ginsenosides have anti-tumor activity. In tumor cells, ginsenosides could induce exogenous apoptosis via enhancing the expression of TRAILs, p53, Fas/FasL, resulting in the activation of caspase cascades (Pro-caspase-8-caspase-3) [61]. Ginsenosides could regulate the JAK/STAT pathway involved in immune regulatory processes. They have regulatory roles on P53, Fas/FasL, and Bax [62]. Indeed, ginsenosides via blocking JAK1/STAT3 could decrease the expression of STAT3 target genes, including survivin, Bcl-2, Bcl-xL. Therefore, they could increase apoptosis in tumor cells via inhibiting the mentioned pathway [62, 63]. Interestingly, they could inhibit NF-кB signaling by regulating Bax/Bcl-2 expression, resulting in the inhibition of angiogenesis [64]. Ginsenosides also via blocking the PI3K/AKT/mTOR could inhibit the proliferation of cancer cells and induce apoptosis [48, 65]. Ginsenosides by decreasing the expression of some cell cycle proteins including Cyclin-B1 could block tumor cell cycles [66, 67]. Ginsenosides by upregulating the expression of LC3-II, Beclin-1, and Atg7 could also induce autophagy, resulting in tumor cell death [68, 69]

20-(s)-ginsenoside Rg3 has been found to reduce the viability of human leukemic cells and induce apoptosis in these cells. Such effects have been accompanied by a significant decrease in the expression of several proteins from the PI3K/AKT cascade. Besides, 20-(s)-Ginsenoside Rg3 has increased activity of caspase-3 and caspase-9. Therefore, this substance enhances apoptosis of human leukemic cells possibly via decreasing expression of PI3K/AKT family proteins (Fig. 3). Besides, induction of caspase-3 and caspase-9 activity mediates induction of apoptosis, suggesting a possible application of this substance for the treatment of leukemia [42]. The anti-angiogenic impact of Ginsenoside Rg3 has also been assessed in patients with acute leukemia. Treatment of bone marrow stromal cells originated from patients with this type of leukemia with Ginsenoside Rg3 has led to inhibition of VEGF and HIF-1α expressions. Moreover, Ginsenoside Rg3 could reduce expressions of HIF-1α and VEGF (Fig. 3) in the serum samples of patients with acute leukemia. Functionally, this substance has reduced phosphorylation of AKT and ERK1/2 in bone marrow stem cells [43].

### Gastrointestinal cancers

Ginsenoside Rh4 has shown a strong anticancer impact in esophageal cancer cells as well as animal models of this cancer. This substance suppresses the growth of cancer cells by arresting cancer cells at the G1 phase (Fig. 3). Moreover, Ginsenoside Rh4 suppresses aerobic glycolysis in this type of cancer by blocking the production of lactate, absorption of glucose, and synthesis of ATP. These effects lead to a reduction of extracellular acidification and oxygen consumption rates. AKT has been suggested as a putative target of Ginsenoside Rh4 through which inhibits aerobic glycolysis. Ginsenoside Rh4 has resulted in the deregulation of AKT, while insulin treatment has abrogated the suppressive impact of Ginsenoside Rh4 on aerobic glycolysis. On the contrary, AKT inhibitors have increased the suppressive impact of Ginsenoside Rh4 on aerobic glycolysis. Based on the results of molecular docking assays, Ginsenoside Rh4 binds to the interdomain region of AKT. Moreover, Ginsenoside Rh4 has decreased levels of PD-L1 through the AKT/mTOR pathway. Therefore, the anticancer impact of Ginsenoside Rh4 in esophageal cancer is exerted through inhibition of aerobic glycolysis and PD-L1 expression [44].

20(S)-Ginsenoside Rg3 has been shown to enhance the anticancer effects of Sorafenib in hepatocellular carcinoma. This kind of treatment has resulted in the up-regulation of levels of PTEN, Bax, and cleaved caspase-3, while down-regulation of levels of phosphorylated PDK1 and phosphorylated Ak3. Notably, in vivo experiments have shown a decrease in tumor volume and weight following administration of the combination of Sorafenib and 20(S)-Ginsenoside Rg3. Therefore, this study has shown the synergism between 20(S)-Ginsenoside Rg3 and Sorafenib in the treatment of hepatocellular carcinoma through modulation of PTEN/AKT signaling [45]. Another study has shown the effects of the combination of CA4P and Ginsenoside Rd on the reduction of HIF-1α expression in hepatocellular carcinoma cells via the PI3K/AKT/mTOR pathway [46]. Table 5 shows the effects of Ginsenoside in the treatment of gastrointestinal cancers.

**Table 5:** Effects of ginsenoside in gastrointestinal cancers

Type of Diseases	Samples	Cell Lines	Ginsenoside	Dose range	Target	Pathway	Function	Ref
Esophageal Cancer	In vitro, in vivo (mice)	Eca109, KYSE150, HET-1 A	Rh4	0-100 µM, 40 mg/kg	PD-L1	AKT/mTOR	Ginsenoside Rh4 via the AKT/mTOR pathway could suppress aerobic glycolysis in Eca109 and KYSE150 cells.	[44]
Hepatocellular Carcinoma (HCC)	In vivo (mouse), In vitro	HepG2, Huh7	20(S)- Rg3	5 mg/kg, 0–300 µg/mL	PTEN, Bax, PDK1, Caspase-3	PTEN/AKT	20(S)-Ginsenoside Rg3 could modulate PTEN/AKT pathway in HCC.	[45]
HCC	In vitro	HepG2	Rd	2–20 µM	HIF-1α	PI3K/AKT, mTOR	CA4P (combining combretastatin A4 phosphate) and ginsenoside Rd via the PI3K/AKT/mTOR pathway could inhibit the expression of HIF-1α in HepG2 cells.	[46]
Liver cancer	In vitro	HepG2	Rh2	0-17.5 µM	Caspase-3/8, Cyclin-D1/D3/E, CDK2	AKT/p38 MAPK	Octyl ester of ginsenoside Rh2via the AKT/p38 MAPK pathway could active apoptosis in HepG2 cells.	[47]
Pancreatic Cancer	In vivo (mouse), In vitro	BxPC-3, AsPC-1	Rg3	3 mg/kg, 0-160 µM	Caspase-3/9, PARP	EGFR/PI3K/AKT	Ginsenoside Rg3 via downregulating the EGFR/PI3K/AKT pathway could enhance erlotinib anti-proliferative activity in pancreatic cancer.	[48]
Colon Cancer	In vitro	SW620, LOVO	Rg3	0–1000 µM	N-cadherin, E-cadherin, MMP-9	PI3K/AKT	Ginsenoside Rg3 via the PI3K/AKT pathway could enhance the function of anticancer effect 5-FU in both colon cancer cells.	[49]

### Gynecologic cancers

Two studies have demonstrated the beneficial effects of Ginsenosides in the treatment of gynecological cancers. First, 20(s)-ginsenoside Rg3 has been shown to reduce viability and induce apoptosis of ovarian cancer cells in a dose- and time-dependent manner. This substance could down-regulate expressions of PI3K/AKT (Fig. 3) and IAP family proteins. Moreover, it could activate caspase-3 and -9 [50]. Another study has demonstrated the effects of Ginsenoside Rh2 in the inhibition of proliferation and migration of cervical cancer cells through modulation of the AKT/GSK-3β axis [51]. Table 6 shows the outlines of these studies.

**Table 6:** Effects of ginsenoside in gynecologic cancers

Type of cancer	Samples	Cell Lines	Ginsenoside	Dose range	Target	Pathway	Function	Ref
Ovarian Cancer (OC)	In vitro	HO-8910	20(S)-Rg3	0-100 µg/mL	Caspase-3/9, Bcl-2, Bax, XIAP, cIAP1/2	PI3K/AKT	20(s)-ginsenoside Rg3 via the PI3K/AKT and XIAP pathways could improve apoptosis in human OC HO-8910 cells.	[50]
Cervical Cancer (CC)	In vitro	HeLa	20(S)-Rh2	10–50 µM	Ncadherin, GSK-3β, Vimentin, Ecadherin, Zeb1, Snail-1	AKT	Rh2 via the AKT/GSK-3β axis could inhibit cell proliferation and migration of HeLa cells.	[51]

### Breast cancer

Ginsenoside Rd has been shown to inhibit VEGF-induced migration, tube formation, and proliferation of HUVEC cells in a dose-dependent manner. Moreover, Ginsenoside Rd could abrogate VEGF-induced emergence of the vessels from aortic rings, and suppress vessel construction in vivo. In both normoxia and hypoxia, Ginsenoside Rd has inhibited VEGF-associated induction of AKT/ mTOR cascade in HUVECs. Intraperitoneal administration of Ginsenoside Rd to xenograft model of breast cancer has resulted in the reduction of tumor volume and weight and decrease in tumor angiogenesis. Moreover, Ginsenoside Rd has suppressed proliferation, enhanced apoptosis inhibited AKT/mTOR/P70S6 kinase cascade in breast cancer [52]. The molecular mechanism of the anti-proliferative and proapoptotic impact of Ginsenosides Rg3 in breast cancer cells has also been explored in a cell line that has constitutive activation of NF-кB and p53 mutation. Ginsenoside Rg3 has suppressed DNA binding and transcriptional activity of NF-кB. These impacts have been exerted through inhibition of IKKβ function, destruction of IκBα, and consequent nuclear translocation of the p65 subunit of NF-кB. Ginsenoside Rg3 has increased apoptosis in MDA-MB-231 cells through suppressing NF-кB cascade via inactivating ERK and AKT (Fig. 3) and destabilizing mutant p53 [53]. Table 7 shows the effects of Ginsenosides in the treatment of breast cancer.

**Table 7:** Effects of ginsenoside in breast cancer

Samples	Cell Lines	Ginsenoside	Dose range	Target	Pathway	Function	Ref
In vivo (Rat, Mouse), In vitro	HUVECs, MDA-MB-231	Rd	1–10 mg/kg, 0–50 µM	HIF-1α, Bax, Bcl-2, Caspase-3, p70S6K	AKT/mTOR	Ginsenoside Rd via regulating the AKT/mTOR/p70S6K axis could suppress breast tumor growth and angiogenesis.	[52]
In vitro	MDA-MB-231	Rg3	0–30 µM	NF-кB, p65, IκBα, IKKβ, p53	ERK, AKT	Ginsenoside Rg3 via the ERK/AKT pathway could affect apoptosis by suppressing the activation of NF-кB in human BCa.	[53]
In vitro	MCF-7	Rk1	0-160 µM	p21, p53, Cyclin-A, CDK2, Bax, Bcl-2, Cytochrome-C, Caspase-3/8/9, PTEN	PI3K/AKT, mTOR	In MCF-7 cells, ginsenoside Rk1 via ROS-mediated PTEN/PI3K/AKT/mTOR pathway could induce cell death.	[54]
In vivo (Mouse)	MCF-7	Rg5	10–20 mg/kg	Caspase-8/9/3, Bax, Cytochrome-C, PARP, Bcl-2	PI3K/AKT	Ginsenoside Rg5 via the PI3K/AKT pathway could induce apoptosis and autophagy in BCa.	[55]

### Brain tumors

Ginsenoside Rh2 has been shown to reduce the viability and proliferation of glioma cells via modulating AKT [56]. Moreover, this substance has decreased the invasiveness of glioblastoma cells in a dose-dependent manner as demonstrated in scratch wound healing and Transwell cell migration assays. Besides, the suppressive impact of Ginsenoside Rh2 on cell migration has been found to be exerted via down-regulation of MMP-13. Ginsenoside Rh2 suppresses the expression of MMP13 via the PI3k/AKT pathway. Therefore, Ginsenoside Rh2 can inhibit migration of glioblastoma via suppressing AKT-associated MMP13 activation [57].

### Other cancers

A recent experiment in osteosarcoma cells has shown that ginsenoside Rh2 significantly suppresses the viability of U20S cells in a dose- and time‐dependent manner, and inhibits their migration. Moreover, the effects of this substance on the induction of apoptosis in U20S cells have been verified through the conduction of TUNEL, DAPI, annexin V/PI, and JC‐1 assays. Ginsenoside Rh2 can also decrease expression of Bcl‐2, caspase 3, and caspase 9, and enhance Bax levels in osteosarcoma cells. Functionally, ginsenoside Rh2 enhances apoptosis of U20S cells through increasing activity of MAPK pathway and suppressing activities of PI3K/AKT/mTOR and NF‐кB pathways in osteosarcoma cells (Fig. 3). Thus, ginsenoside Rh2 exerts anticancer effects in osteosarcoma through influencing the activity of MAPK, PI3K/AKT/mTOR, and NF‐кB pathways [58]. Ginsenoside Rg3 via inhibiting the PI3K/AKT pathway could exert antitumor effects in lung cancer [59]. Finally, Ginsenoside Rg3 via ERK and AKT pathways could inhibit angiogenesis of melanoma and inhibit the growth of B16 cells [60]. Table 8 shows the effects of Ginsenosides in diverse cancers.

**Table 8:** Effects of Ginsenoside in diverse cancers

Type of cancer	Samples	Cell Lines	Ginsenoside	Dose range	Target	Pathway	Function	Ref
Osteosarcoma (OS)	In vitro	U20S	Rh2	8–80 µM	Bcl-2, Bax, NF-кB, Caspase-3/9	PI3K/AKT/mTOR, MAPK	Ginsenoside Rh2 via NF-кB, MAPK, and PI3K/AKT/mTOR pathways could suppress proliferation and migration in U20S cells.	[58]
Lung Cancer	In vivo (Mouse), In vitro	A549, H23	Rg3	20 mg/kg, 0-200 µM	-	PI3K/AKT	Ginsenoside Rg3 via inhibiting the PI3K/AKT pathway could exert antitumor effects in lung cancer.	[59]
Advanced Metastatic Melanoma	In vitro, In vivo (Mouse)	B16	Rg3	1–15 µg/mL, 0.3-3 mg/kg	VEGF, HIF-1α, MMP-2/9	ERK/AKT	Ginsenoside Rg3 via ERK and AKT pathways could inhibit the angiogenesis of melanoma and inhibit the growth of B16 cells.	[60]
-	In vitro	SW620, LS513, OVCAR8-DXR, A549-DXR	Rp1	0–5 µM, 0–30 µM,	SIRT1, PARP	AKT	A combination of ginsenoside Rp1 and actinomycin D via the AKT/SIRT1 axis could reduce drug resistance.	[70]

## Discussion

Ginsenosides are a group of substances extracted from plants. Although they have been used in traditional medicine for a long time, the underlying mechanisms of their therapeutic effects are being illustrated just recently. Diabetes and CNS disorders are two types of disorders in which the therapeutic effects of Ginsenosides are extensively appraised. The PI3K/AKT signaling has been shown to be the most appreciated target of different Ginsenosides. The beneficial effects of these substances in the treatment of diabetic complications, SCI, cerebral/myocardial ischemia, and several other non-neoplastic conditions are exerted through activating this pathway. On the other hand, experiments in diverse cancer cell lines have shown the inhibitory effects of Ginsenosides on the PI3K/AKT pathway.

HIF-1α, Bax, Bcl-2, Caspase-3/8/9, p70S6K, NF-кB, p65, IκBα, IKKβ, p53, p21, Cyclin-A, CDK2, Cytochrome C and PTEN are other molecules whose expressions are affected by Ginsenosides.

In addition to the observed controversy about the effect of Ginsenosides on the PI3K/AKT signaling, the effects of Ginsenosides on the expression of HIF-1α and VEGF are controversial. Ginsenoside Re has been shown to enhance the expression of HIF-1α in the cytoplasm but reduce its nuclear levels [5]. Moreover, Ginsenoside Rg1 has been reported to increase the expression of HIF-1α [26]. On the other hand, treatment of leukemic bone marrow stromal cells with Ginsenoside Rg3 has led to inhibition of VEGF and HIF-1α expressions [43]. The possible impact of the underlying pathological conditions and different effects of various Ginsenosides or even different doses of these substances on the expression of genes should be assessed in future studies. In addition to the regulatory effects of Ginsenosides on the activity of cancer-related pathways, suppression of aerobic glycolysis by these substances can be regarded as a possible route of anticancer effects of these substances. Moreover, Ginsenosides can enhance the activity of other anticancer drugs including both herbal medicines and targeted therapeutic options on tumor cells indicating their synergisms with a wide range of therapeutic modalities. A combination of Ginsenosides with other anticancer drugs might also reduce resistance of cancer cells to cytotoxic effects of these drugs.

## Conclusion

The above-mentioned studies have mostly assessed the effects of Ginsenosides in cell lines or animal models, lacking evidence from human subjects. Conduction of well-designed studies in human subjects is required for the identification of the proper dose of Ginsenosides in each pathologic condition. Moreover, future studies should identify appropriate markers for the prediction of the response of cancer cells to Ginsenosides.

Finally, based on the observed effects of Ginsenosides on the production of ROS, these agents may be regarded as preventive strategies against the initiation of cancer. However, this field has been little explored by researchers.

## Data Availability

Data sharing not applicable to this article as no datasets were generated or analysed during the current study.
